# Sir Napier Burnett's Report on the Voluntary Hospitals outside London
*Third Annual Report on the Voluntary Hospitals in Great Britain (excluding London) for the Year 1921. By Sir Napier Burnett, K.B.E., M.D., F.R.C.S., F.R.C.P., Director of Hospital Services, Joint Council of the Order of St. John and the British Red Cross Society. With an Introductory Note by the Hon. Sir Arthur Stanley, G.B.E., C.B., M.V.O., Chairman of the Joint Council. Price 2s. 6d.


**Published:** 1923-01

**Authors:** 


### SIR NAPIER BURNETT'S REPORT ON THE VOLUNTARY HOSPITALS OUTSIDE LONDON.*

WE have already in a previous issue made reference to Sir Napier Burnett's able and interesting Report on the position of the Voluntary Hospitals outside London, and doubtless every hospital will have provided itself with a copy. Every one of our readers who, though not directly concerned with hospital administration, is interested in, or has occasion to refer to, statistics of hospital work will find this book invaluable.

The hospitals in the London area, being under the supervision of King Edward's Hospital Fund, are not included in the Report, the object of which is to present to the public detailed information as to the financial position of the voluntary hospital system, and to assist hospital governors and administrators with information whereby they may ascertain how their own hospital position compares with the group averages of other comparable institutions.

**Number of Patients Treated and the Cost.**—Taking Great Britain as a whole (excluding London) details of patients were received for hospitals with 95 per cent. of the total possible beds which show that 2,545,055 individual patients were treated during 1921 at a total cost to the hospitals of £5,275,176. Towards this expenditure the hospitals received £4,854,661, leaving a deficit on the year's working of £420,515. One-third of the total ordinary income of the hospitals was derived from workmen's contributions and patients' payments.

**Hospital Provision for Patients of Moderate Means.**—Sir Napier Burnett considers that this is probably the most urgent problem calling for solution in the hospital world. The hospital of to-day is a much more expensive institution than its predecessor of even twenty years ago, in large measure because of the many advances that have been made in scientific medicine and surgery during those years, and to keep abreast of these developments elaborate and expensive apparatus is required. New departments have had to be established to meet the demands of specialisation. All this new apparatus leads to a greater precision in the diagnosis and treatment of disease. The benefits of these scientific attainments are now available for the poor in our public hospitals and for the rich in private hospitals, but those of moderate means who are debarred from the private institution because of the cost, and yet are above the income limit for admission into the public hospital, suffer a real hardship in being unable to participate in the benefits of modern hospital scientific resources.

The solution, says Sir Napier Burnett, will not be reached until the medical profession come to realise that the payment of a hospital medical staff is really centred in the question of the provision of hospital beds for this class of the community, and he appeals to the profession to take this matter into consideration and act in an advisory capacity to those hospital governors and trustees who are seeking the solution of this problem.

**Relative Hospital Statistics.**—Care is needed in studying hospital statistics, the relative number of beds being no sufficient criterion. For instance, two hospitals with the same number of beds may show in their annual reports issued to the public that one has cost 50 per cent. more than the other, and the impression produced when they both appeal to the public for support may be that the one hospital is extravagant and inefficiently administered as compared with the other. Whereas the true position may be that the hospital showing the higher cost is really the more economical in that it is supplying a much fuller service to the public than its neighbour.

Again, the teaching hospitals (the figures, it will be remembered, exclude London) are in number only 3 per cent. of the whole, but their available beds are 19 per cent. of the total, and they, in fact, treated 28 per cent. of all the patients.

The following figures show the relative importance of the teaching hospitals to the other hospitals throughout the provinces :—

| | No. of hospitals. | No. of available beds. |
|---|---|---|
| England .. .. | 581 | .. 33,356 |
| Scotland .. .. | 75 | .. 6,887 |
| Total .. | 656 | .. 40,243 |
| Teaching hospitals | 19 | .. 7,772 |

Further, while the total deficit incurred by the 656 hospitals during the year was £419,138, the 19 teaching hospitals were responsible for a deficit of £262,644, or 62.66 per cent. of the total deficit, thus leaving a net deficit for 1921 of £156,494 incurred by 637 hospitals.

These figures, Sir Napier Burnett points out, lend support to the claim for special consideration for these hospitals as a specific group, worthy of assistance from the National Exchequer, in that their research and educational work is of importance to a much wider community than that represented in the limited area in which a teaching hospital is situate.

The figures given are those for which reports were received in time for inclusion in the report. The complete figures, including several small or cottage hospitals, are given as :—

| | Total possible number of hospitals. | Total available beds. |
|---|---|---|
| England and Wales | 641 | .. 34,913 |
| Scotland .. .. | 90 | .. 7,290 |
| Total .. | 731 | .. 42,203 |

Our readers will find a mass of information as to the classification of patients, the numbers of operations, analyses of income and expenditure, &c., in the graphs and tabular statements clearly set out in the hundred pages of this illuminating document.

* Third Annual Report on the Voluntary Hospitals in Great Britain (excluding London) for the Year 1921. By Sir Napier Burnett, K.B.E., M.D., F.R.C.S., F.R.C.P., Director of Hospital Services, Joint Council of the Order of St. John and the British Red Cross Society. With an Introductory Note by the Hon. Sir Arthur Stanley, G.B.E., C.B., M.V.O., Chairman of the Joint Council. Price 2s. 6d.

